# Using ecosystem health and welfare assessments to determine impacts of wild collection for public aquariums

**DOI:** 10.1371/journal.pone.0285198

**Published:** 2023-05-02

**Authors:** Brittany Fischer, Jessica Pempek, Kelly Ann George, Jaylene Flint, Thomas Wittum, Mark Flint

**Affiliations:** 1 Department of Veterinary Preventive Medicine, College of Veterinary Medicine, The Ohio State University, Columbus, Ohio, United States of America; 2 Department of Animal Sciences, College of Food, Agricultural, and Environmental Sciences, The Ohio State University, Columbus, Ohio, United States of America; CIFRI: Central Inland Fisheries Research Institute, INDIA

## Abstract

Aquatic ecosystems are currently facing a multitude of stressors from anthropogenic impacts, including climate change, pollution, and overfishing. Public aquariums positively contribute to ecosystems through conservation, education, and scientific advancement; but may also negatively detract from these systems through collection of animals from the wild and sourcing from commercial suppliers. Changes within the industry have occurred, although evidence-based assessments of 1) how aquariums collect and maintain their populations to determine sustainability of the environment they have harvested; and 2) the welfare of these harvested animals once within the aquariums are still needed. The objectives of this study were to assess the ecosystem health of locations aquariums frequently visit to collect fish from the wild, and then evaluate the wellbeing of fishes at aquariums after extended periods in captivity. Assessments included use of chemical, physical, and biological indicators at field sites, and use of a quantitative welfare assessment at aquariums for comparison to species reared through aquaculture. Anthropogenic pressures at field sites were observed, but no evidence of high degradation or compromised health of animals were found. Welfare assessments of aquarium exhibit tanks produced high-positive scores overall (> 70/84), demonstrating that both wild collected (avg. score 78.8) and aquaculture fishes (avg. score 74.5) were coping appropriately within their environments. Although findings indicated that fish can be taken from the wild at low-moderate rates without any deleterious impact on the environment and cope equally well in aquarium settings, alternatives such as aquaculture should be considered as a strategy to reduce pressure on known stressed aquatic environments or where significant numbers of fishes are being taken.

## Introduction

Coastal environments are some of the most vulnerable ecosystems to anthropogenic stressors yet over 50% of the United States population lives within 50 miles of these areas [1]. Examples of human imposed stressors include impacts from climate change such as sea level rise, beach erosion, and flooding, point and non-point pollution from agricultural, industrial, and urban runoff, and biodiversity loss of important species through overfishing [2–6]. In response to better understand the metrics of these pressures, concepts, such as ecosystem health, have evolved as frameworks to consider these stressors holistically and address complex issues influencing humans, animals, and the environment [7].

Good ecosystem health has been defined as the condition of an entire system that is able to sustain services to humans, maintain resilience to natural and human imposed pressures, as well as recognizes human, animal, and environmental wellbeing and their interdependence on one another [7]. To determine the ecosystem health of an environment, it is now recognized that the use of biological, physical, and chemical indicators must be employed [8–11]. Reference information is often incorporated for comparison of potentially degraded ecosystems to their historical natural range of variability with considerations for ecological successes as well as stakeholder inputs [12]. Fish are often used as sentinel species within ecosystem health studies, as they reside in almost all aquatic environments and are of ecological and economic importance due to their roles in the food web, ecotourism, as well as recreational and commercial activities [3, 13]. Examples of other indicators often used in aquatic ecosystem health studies include measurements of water quality and environmental contaminants as chemical indicators, as well as physical indicators such as land use and channel morphology [reviewed by 14].

Commercial fisheries have adopted this ecosystem health approach through cultural and legislative shifts from single species to ecosystem-based fisheries management, recognizing the services and societal benefits of the industry, as well as the direct and indirect pressures humans impose when harvesting from these environments [15, 16]. Although advances in commercial fishing have occurred, recreational fishing restrictions are limited to certain species, and completion of catch surveys are recommended but not required [17]. Further, the public aquarium industry regularly collects fishes from the wild bringing the potential for additional stress on vulnerable environments that animals are collected from [18].

Programs such as Species Survival Plans have been developed by accrediting organizations for zoological institutions, including public aquariums, to advance sustainable population management initiatives [19]. To date, most of the developed programs are limited to terrestrial and charismatic aquatic species leading to little to no collection of these animals from the wild [20]. For aquariums, previous challenges of husbandry requirements for rearing fishes in intensive larval programs have led to most species being collected from the wild or purchased from commercial suppliers [19, 21]. Although most freshwater species are now available through commercial aquaculture, around 95% of marine fish species are still sourced from the wild for private home aquariums and public displays at large institutions [22–25]. As of 2019, the Association of Zoos and Aquariums (AZA) established the Aquatic Collections Sustainability Committee to develop population guidelines for their over 230 member institutions to “assure thriving, sustainable, aquatic populations” [26]. These institutions have also demonstrated interest in increasing marine species available through aquaculture, although more resources are needed [27, 28].

Along with shifts towards sustainable management, zoological institutions now also prioritize animal wellbeing within their institutions with the goal to become the gold standard for animal welfare centered care of animals [29]. Members of accrediting organizations are required to perform annual welfare assessments of animals in their collections to maintain accreditation, developed through influence of previously established animal welfare frameworks [for frameworks see 30–32]. Continuous monitoring of species in aquariums is especially important as exhibit tanks are often structurally and socially complex to be eye-catching and frequently contain predator and prey animals sharing a new restricted environment. Previous studies reviewing the literature have identified the lack of animal welfare research of fish species in zoological systems, creating a gap within the literature of how these species cope within captive environments [33–35]. Additionally, concerns for and lack of understanding regarding animal wellbeing in aquaculture settings has been identified as an area in need of additional consideration [28].

The objectives of this study were to 1) assess the ecosystem health of locations aquariums frequently visit to collect fish from the wild compared to baseline reference parameters; and then 2) evaluate the wellbeing of the wild collected fishes in exhibit tanks at aquariums compared to similar fish species reared through aquaculture. Hypotheses included: 1) reference water parameters would not be different than field data collected at aquarium sites; 2) species diversity at aquarium collection sites would be lower than baseline reference information; and 3) total welfare assessment scores of aquaculture exhibit tanks would be greater than that of wild collected exhibit tanks.

## Materials and methods

Sampling and handling of animals occurred according to acquired South Carolina (5762) and North Carolina (2026400) permits and approved by The Ohio State University Institutional Animal Care and Use Committee (IACUC) protocol (2021A00000040). Seine nets were used for sampling animals, detailed below, and no protected species were sampled. Animals selected for health assessments were mildly sedated through emersion in tricaine methanesulfonate (MS-222) buffered solution, detailed below. Upon completion of assessments, animals were placed in clean water, monitored, and returned to their respective water bodies after returning to homeostasis (maximum time in human care averaged two hours). No euthanasia of animals occurred.

### Field site descriptions

**Fig 1:**
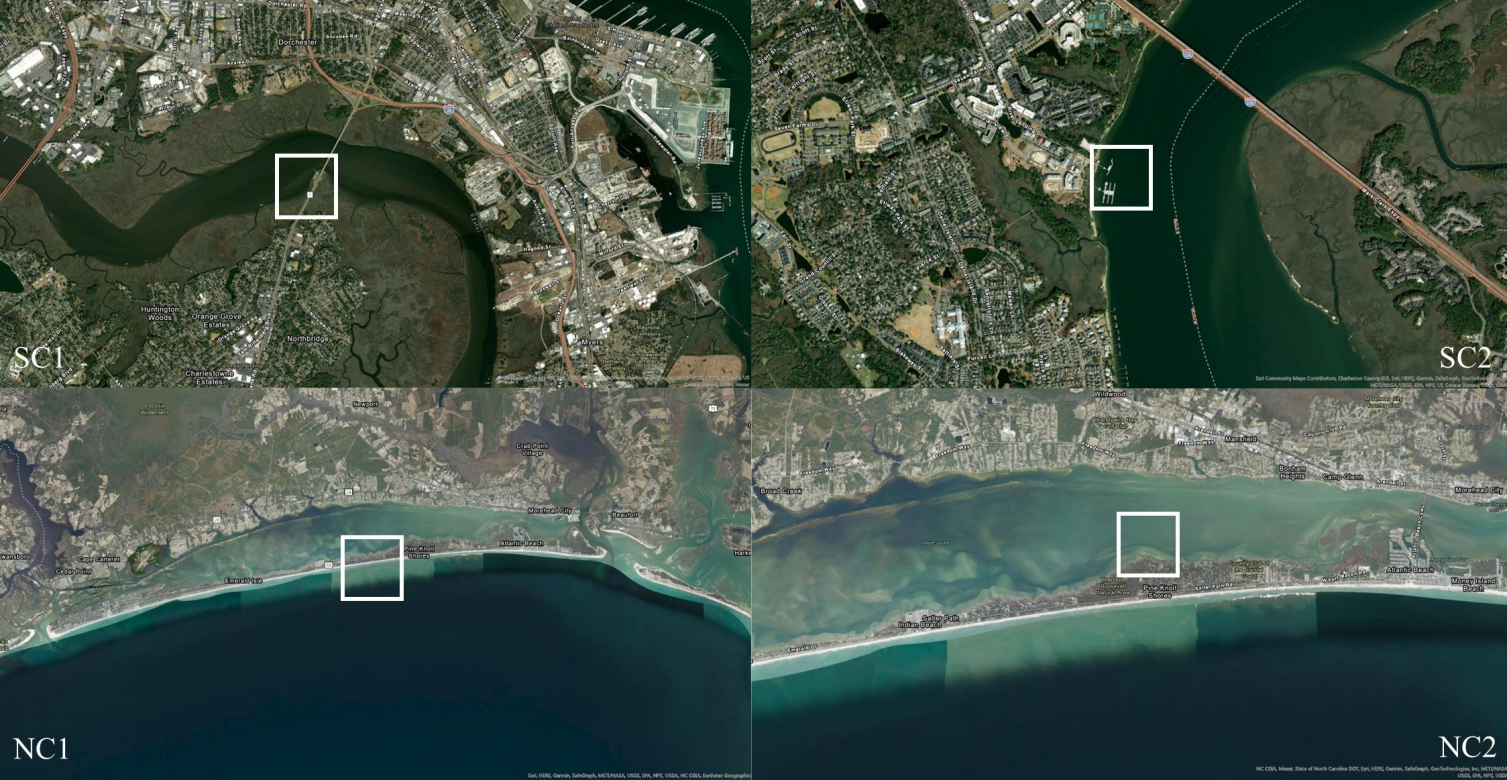
Field site locations. Maps of the four field sites, SC1 (32.834752, - 79.986940), SC2 (32.856259, - 79.902133), NC1 (34.692179, - 76.829056), and NC2 (34.701380, - 76.832141), from where two public aquariums collect species from for their institutions. Maps were created using ArcGIS. Contributors for the SC maps included Esri Community Maps Contributors, City of Charleston, Charleston County GIS, OpenStreetMap, Microsoft, Esri, HERE, Garmin, SafeGraph, GeoTechnologies, Inc., METI/NASA, USGS, EPA, NPS, US Census Bureau, USDA, and Maxar. Contributors for the NC maps included Esri Community Maps Contributors, State of North Carolina DOT, Esri, HERE, Garmin, SafeGraph, GeoTechnologies, Inc., METI/NASA, USGS, EPA, NPS, Census Bureau, USDA, NC CGIA, and Maxar.

Assessments occurred at four sites, two (SC1 and SC2) in Charleston, South Carolina (32.834752, - 79.986940 respectively and 32.856259, - 79.902133 respectively), and two (NC1 and NC2) in Pine Knoll Shores, North Carolina (34.692179, - 76.829056 respectively and 34.701380, - 76.832141 respectively) as locations that two public aquariums visit on an annual basis to collect animals from the wild for their institutions (Fig 1).

Both locations in Charleston, SC were estuarine ecosystems with public access. SC1 was located at Northbridge Park, established in 2014, on the Ashley River under the Cosgrove Bridge. A long-time fishing location for locals before officially becoming a Division of Transportation managed park, the area now includes a dock, pier, and a kayak and canoe launching point [36]. SC2 was located on Daniel Island, near the Wando River Bridge at Waterfront Park. Urban development from agricultural land began in this area in the 1990s and the area now includes a shopping area, apartments, and the Charleston boating club [37]. Oyster restoration led by the Division of Natural Resources South Carolina Oyster Recycling and Enhancement Group began in 2010 and is ongoing [38]. Installment of a bulkhead retaining wall at this site occurred in 2022.

NC1 was an Atlantic Ocean facing location at Iron Steamer Public Beach. This site once included a fishing pier, which was damaged by a hurricane, torn down in 2004, and replaced by housing [39]. At the time of sampling, beach replenishment programs were ongoing and included annual stocking of sand and planting of American beach grass (*Ammophila breviligulata*) [40]. NC2 was located within Bogue Sound in a protected area behind the aquarium, with fishing prohibited except for North Carolina Aquariums. Active oyster restoration was also observed at this location, led by the North Carolina Coastal Federation [41].

### Reference site descriptions

Historical catch per unit effort species data and water chemistry data (1990’s–present) were obtained from North and South Carolina agencies to be compared to field site data, when applicable (Table 1). For the South Carolina locations, data was obtained from the South Carolina Department of Natural Resources Trammel Net (182.88 m x 2.44 m) Survey. Two stations were selected for each site based on proximity to aquarium collection sites. For the North Carolina locations, data was obtained from the North Carolina Division of Marine Fisheries Program 120 Estuarine Otter Trawl (1.83 m x 9.14 m) Surveys. Two stations were selected for comparison to NC2 as similar inlet ecosystems, within Bogue Sound, with consistent historical data. No reference data was appropriate for comparison to NC1. Reference data were grouped into four periods to test for decadal differences of diversity of species and differences of water temperature, salinity, and dissolved oxygen (Table 1).

**Table 1:** Reference station information and periods of data collection.

State agency	Reference stations	Period	N sampling days	N fish sampled	Field site comparison
South Carolina DNR	Ashley River: AR12 and AR29	1 (≤ 1999)	51	1560	SC1
		2 (2000–2009)	106	1141	
		3 (2010–2019)	110	1513	
		4 (≥ 2020)	16	335	
South Carolina DNR	Wando River left: LW09 and LW10	1 (≤ 1999)	64	939	SC2
		2 (2000–2009)	80	844	
		3 (2010–2019)	83	538	
		4 (≥ 2020)	8	57	
North Carolina DMF	Bogue Sound: Goose Creek (CC21) & Taylor Bay (CC21A)	1 (≤ 1999)	52	18046	NC2
		2 (2000–2009)	36	31903	
		3 (2010–2019)	39	34723	
		4 (≥ 2020)	8	3264	

### Ecosystem health assessments of field sites

Ecosystem health was measured at field sites and included water and soil chemistry, habitat complexity and quality, population diversity sampling, and health evaluations of fishes. Data collection occurred at North and South Carolina field sites over three periods from spring 2021-summer 2022 (Table 2). Reference information was used for comparison to field sites instead of a control site due to inability to sample a true no take area, absent of all commercial and recreational fishing. Data were compared to reference information to test for decadal differences of water parameters and species diversity at these sites as a proxy for environmental change. Indicators of ecosystem health were grouped into chemical, physical, and biological indicators.

**Table 2:** Field site populations and data collection timeline.

Site	Period	*n* sampling days	*n* fish sampled	*n* skin, fin, gills collected	*n* blood collected	*n* water samples	*n* soil samples	*n* EA performed
SC1	1	2 (May 2021)	1291	25	.	2	.	.
	2	2 (Sept. 2021)	565	.	.	2	.	.
	3	7 (June 2022)	1361	52	31	7	7	7
SC2	1	2 (May 2021)	410	25	.	2	.	.
	2	2 (Sept. 2021)	20	.	.	2	.	.
	3	7 (June 2022)	1577	73	60	7	7	7
NC1	1	2 (May 2021)	577	11	.	2	.	.
	2	2 (Sept. 2021)	153	.	.	2	.	.
	3	7 (June 2022)	6337	57	39	7	7	7
NC2	1	2 (May 2021)	582	8	.	2	.	.
	2	2 (Sept. 2021)	645	.	.	2	.	.
	3	7 (June 2022)	7923	80	70	7	7	7

Chemical indicators of ecosystem health included water parameters and soil chemistry. Water quality data was collected using an In-Situ SmarTROLL™ Multiparameter Handheld Water Quality Meter. Temperature, pH, dissolved oxygen, salinity, and conductivity parameters were collected each time sampling occurred (*n* = 11 readings per site). Soil chemistries were collected in period three (*n* = 7 samples per site) and included low-high readings of nitrogen (18 kg, 73 kg, or 145 kg/ A/15.25 cm soil), phosphorus (4 kg, 9 kg, or 29 kg/ A/15.24 cm soil), and potassium (18 kg, 36 kg, or 73 kg/ A/15.24 cm soil). Samples were collected using the grab method and a Soil Quality Test Kit [42, 43].

Physical indicators of ecosystem health were ranked 1–5 (poor-excellent) within the categories of habitat complexity and morphology, discharges, recreational and commercial disturbances, and coastline development using a modified environmental impact assessment based on previously established habitat quality index tools [9, 44–46]. Rankings were assigned to indicators based on observed percentage of impacted area at each site with the highest achievable total score = 50. Sites were scored each sampling day by the same individual during period three (*n* = 7 assessments per site).

Fishes, sentinel species of ecosystem health, were selected as the biological indicators. Surveying occurred using seine nets (1.22 m x 6.1 m and 1.83 m x 9.14 m) for a total of two hours per site each sampling day (*n* = 11 sampling days per site). Individuals were identified at the species level, total lengths measured, and then returned to the water body they were collected from. Animals were grouped into functional guilds based on their place within the water column and prey species consumed (Table 3) [47, 48]. A subpopulation of surveyed species was selected for full health assessments including blood collection and skin, fin, and gill sample collection for examination of parasitic loads under an onsite microscope. Animals were selected based on size appropriateness for sample collection.

**Table 3:** Functional guild descriptions.

Water column	Description	Functional guild
Benthivores	Consumes prey associated with bottom substrate	1: Generalist
		2: Detritus only
Epifaunal	Consumes organisms associated with eelgrass, algae, and near bottom habitats	3: Crustacivores (crustaceans)
		4: Planktivores (plankton)
		5: Algae
Pelagic	Consumes organisms mainly in the water column	6: Planktivores
		7: Detritus
		8: Crustacivores
		9: Piscivores (fish)

Sedation of fish occurred through emersion in < 50 mg/L MS-222 buffered solution for skin, fin, and gill sample collection (*n* = 331 total individuals sampled). Using iris surgical scissors, fin and gill clips were collected, and gill samples totaled 2–3 tips of lamellae per fish. Skin scrapes were obtained by lightly sliding a microscope coverslip along the lateral surface of the fish for collection of the mucus layer. All samples were examined under an onsite field microscope immediately after collection and rated using the 0–3 (none–many) veterinary technique [technique described in 49].

One blood sample (< 0.05 mL), collected from the caudal vertebrae, was obtained from a subset of individuals selected for skin, fin, and gill collection (*n* = 200 total individuals sampled). A lateral approach was employed between the scales for creation of a blood smear. Staining of blood smears occurred using Standard H&E. Estimates of total white blood cell counts occurred through taking the sum of 10 high powered fields and multiplying by 200 [protocol described in 50]. Differential leukocyte counts were obtained by counting 100 white blood cells, and included lymphocytes, neutrophils, basophils, monocytes, and eosinophils [described in 51, 52].

### Welfare assessments

Wellbeing of fishes within exhibit tanks at aquariums was evaluated semi-annually (*n* = 16 total assessments) over the same three periods using a modified welfare assessment influenced by the Five Domains Model [welfare assessments described in 32 and 53]. Indicators in the areas of nutrition, physical environment, health, and behavioral interactions were scored from 1–3 (high risk- good). Scores were assigned based on observations of animals and environmental parameters, as well as obtained health and nutrition information from staff members. Assessments included scoring of resource-based indicators, such as appropriate diet, environmental complexity, and preventive care, as well as animal indicators through observed evidence of species appropriate behaviors, aggression and/or agnostic behaviors, and active avoidance of animals to other individuals within exhibit tanks as well as in areas where interactions with visitors occurred. Behavioral indicators were weighted by two. Indicator scores were summed at the completion of each assessment and the highest achievable assessment score = 84. Assessments were conducted by the same individual in front of exhibits containing species whose natural ranges were the same as the species surveyed at field collection sites.

### Statistical analysis

Data were analyzed using lme4, mclogit, vegan, and tidyverse packages in R Statistical Software v 4.2.0 [54–57]. Assumptions of data were verified for each model using residual vs fitted and q-q plots. Statistically significant values were those *p* < 0.05 and biologically significant *p* < 0.08.

Reference data, grouped into four periods, was first analyzed using Linear Mixed-Effects Models to test for decadal differences at Ashely River (AR), Wando River left (LW), and Bogue Sound (CC) locations and then later each period compared to data collected at the field study sites (Table 1; detailed below). Period (one–four) was used as the fixed effect and location the random effect to account for repeated sampling and similarities within sites. Shannon-Wiener diversity indexes, temperature, salinity, and dissolved oxygen were run as the dependent variables in separate models for AR, LW, and CC sites.

Water and soil chemistries were examined using descriptive statistics and water data for SC1, SC2, and NC2 compared to reference information using Linear Mixed-Effects Models. Temperature, salinity, and dissolved oxygen were run separately as dependent variables and type (reference period one, reference period two, reference period three, reference period four and field data) was the fixed effect. Location was used as the random effect in each model to account for repeated sampling at sites. Separate models were used for each aquarium collection site (SC1, SC2, and NC2).

Environmental assessment data was analyzed using an analysis of variance model. Field site scores for each category were summed and the total score used as the dependent variable. Location (SC1, SC2, NC1, and NC2) was the independent variable to compare sites to one another. *Post-hoc* comparisons of sites occurred using the Tukey method.

Shannon-Wiener diversity indexes for sites SC1, SC2, and NC2 were calculated and then compared to reference locations using Linear Mixed-Effects Models. Type was used as the fixed effect to test for differences between field sites and their respected reference site periods. Location was the random effect to account for repeated sampling and similarities within sites and diversity indexes the dependent variable run in separate models for each site. Pearson’s chi-squared portions tests were used to compare the proportions of animals within each functional guild of field and reference site populations.

Skin, fin, and gill data were analyzed using multinomial logistic regression. ‘None’ was used as the baseline category due to being the most frequent rating when examining samples. Skin, fin, and gill parasites were the dependent variables in separate models and location was used as fixed effect to compare field sites to one another. Blood data was analyzed using analysis of variance models. Dependent variables included estimated total white blood cell counts and each differential white blood cell type. Each were analyzed using separate models and location was used as the fixed effect.

For the welfare assessments, weights were assigned to behavioral indicators by multiplying scores within the behavior category by two. Indicator scores were summed, and totals were used as the dependent variable and period, aquarium, and exhibit were the fixed effects in an analysis of variance model. A separate model was run to test for an effect of origin (wild collected or aquaculture) on total welfare score of exhibits at the North Carolina Aquarium at Pine Knoll Shores due to this institution having both wild collected and aquaculture exhibit tanks.

## Results

### Ecosystem health assessments

Salinity differed by period at all reference sites (AR *p* < 0.01, LW *p* < 0.01, CC *p* = 0.05). Dissolved oxygen was found to differ by period at AR sites (*p* = 0.01) but not at LW or CC sites (Table 4). No differences of temperature across reference periods of sites were found. For SC1 and AR reference periods, no differences of salinity, temperature, or dissolved oxygen were found. Salinity of SC2 was different than LW reference period one (*p* < 0.01) and temperature was different than LW reference periods one-three (*p* < 0.01). No differences of dissolved oxygen at SC2 and reference periods were found. For NC2, salinity was different than CC reference periods one, two, and four (*p* < 0.01) but not three. No difference of dissolved oxygen or temperature was found for NC2 and CC reference periods (Table 4).

**Table 4:** Reference and field site mean ± SD water parameters.

Site	Period	Avg. ± SD Salinity	Avg. ± SD Temperature	Avg. ± SD DO
AR reference	1 (≤ 1999)	16.95 ± 6.04	19.22 ± 8.99	12.04 ± 7.52
	2 (2000–2009)	21.41 ± 5.67	15.27 ± 9.42	13 ± 8.09
	3 (2010–2019)	20.39 ± 6.06	14.13 ± 9.25	12.39 ± 7.52
	4 (≥ 2020)	19.02 ± 5.42	18.76 ± 9.52	9 ± 5.39
SC1 field site	4 (≥ 2020)	17.04 ± 5.21	29.3 ± 3.78	5.6 ± 0.76
LW reference	1 (≤ 1999)	19.4 ± 2.84^a^	12.04 ± 6.97	7.31 ± 1.47
	2 (2000–2009)	23.54 ± 2.55^a^	21.53 ± 6.6	6.55 ± 1.65
	3 (2010–2019)	22.24 ± 2.92^a^	21.44 ± 7.1	6.52 ± 1.88
	4 (≥ 2020)	22.35 ± 1.66	20.25 ± 6.2	9.8 ± 2.23
SC2 field site	4 (≥ 2020)	23.38 ± 1.41	27.66 ± 3.62	6.66 ± 0.85
CC reference	1 (≤ 1999)	31.08 ± 4.58^a^	23.96 ± 4.6	5.68 ± 0.74
	2 (2000–2009)	31.62 ± 5.85^a^	23.87 ± 4.26	6.1 ± 1.45
	3 (2010–2019)	32.55 ± 4.42	25.68 ± 3.27	5.82 ± 1.51
	4 (≥ 2020)	27.38 ± 4.53^a^	23.91 ± 1.34	5.1 ± 1.14
NC1 field site	4 (≥ 2020)	36.65 ± 1.75	24.31 ± 2.71	7.03 ± 0.52
NC2 field site	4 (≥ 2020)	36.65 ± 3.28	28.1 ± 2.81	8.66 ± 1.15

^a^Indicates significant difference between reference period and respected field site

For soil chemistries, average readings with standard deviations included fell within the low range for nitrogen and phosphorus concentrations across all sites except for NC2 (mean phosphorus = 1.14 ± 0.38). Mean potassium levels were medium for SC1 (2.29 ± 0.95) and SC2 (2.43 ± 0.79), low for NC1 (1 ± 0), and high for NC2 (3 ± 0).

**Fig 2:**
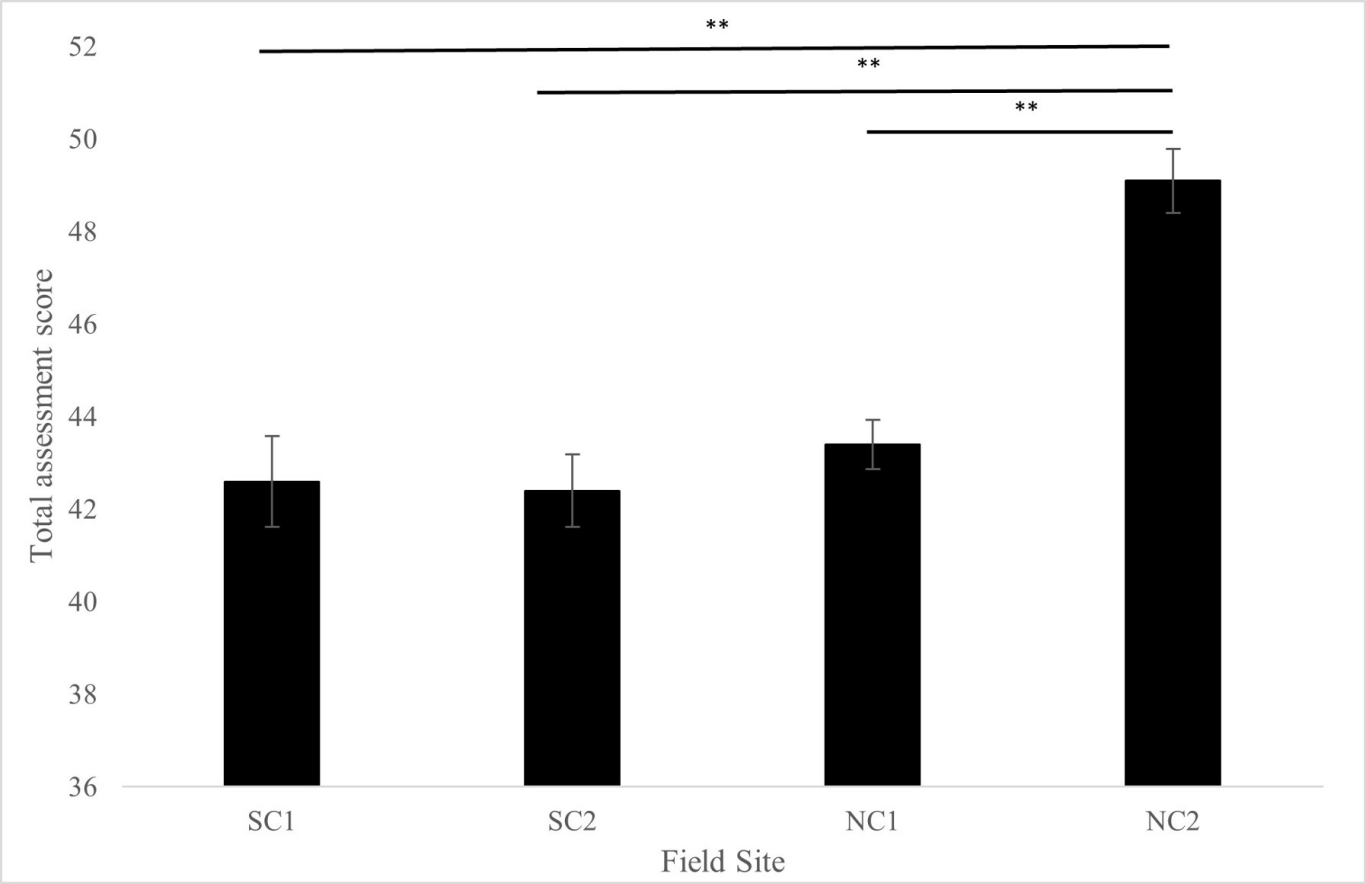
Average field site total environmental assessment score. Influence of field site on average total environmental assessment score (*p* < 0.01). Scores calculated by summing ratings 1–5 (poor–excellent) in all categories, highest achievable score = 50. **indicates *p* < 0.01 after *post-hoc* comparisons.

Field site location was found to influence environmental assessment score (*p* < 0.01). *Post-hoc* comparisons of field sites to one another resulted in differences between NC2 and all other sites. No other differences of total score between sites were found (Fig 2).

**Fig 3:**
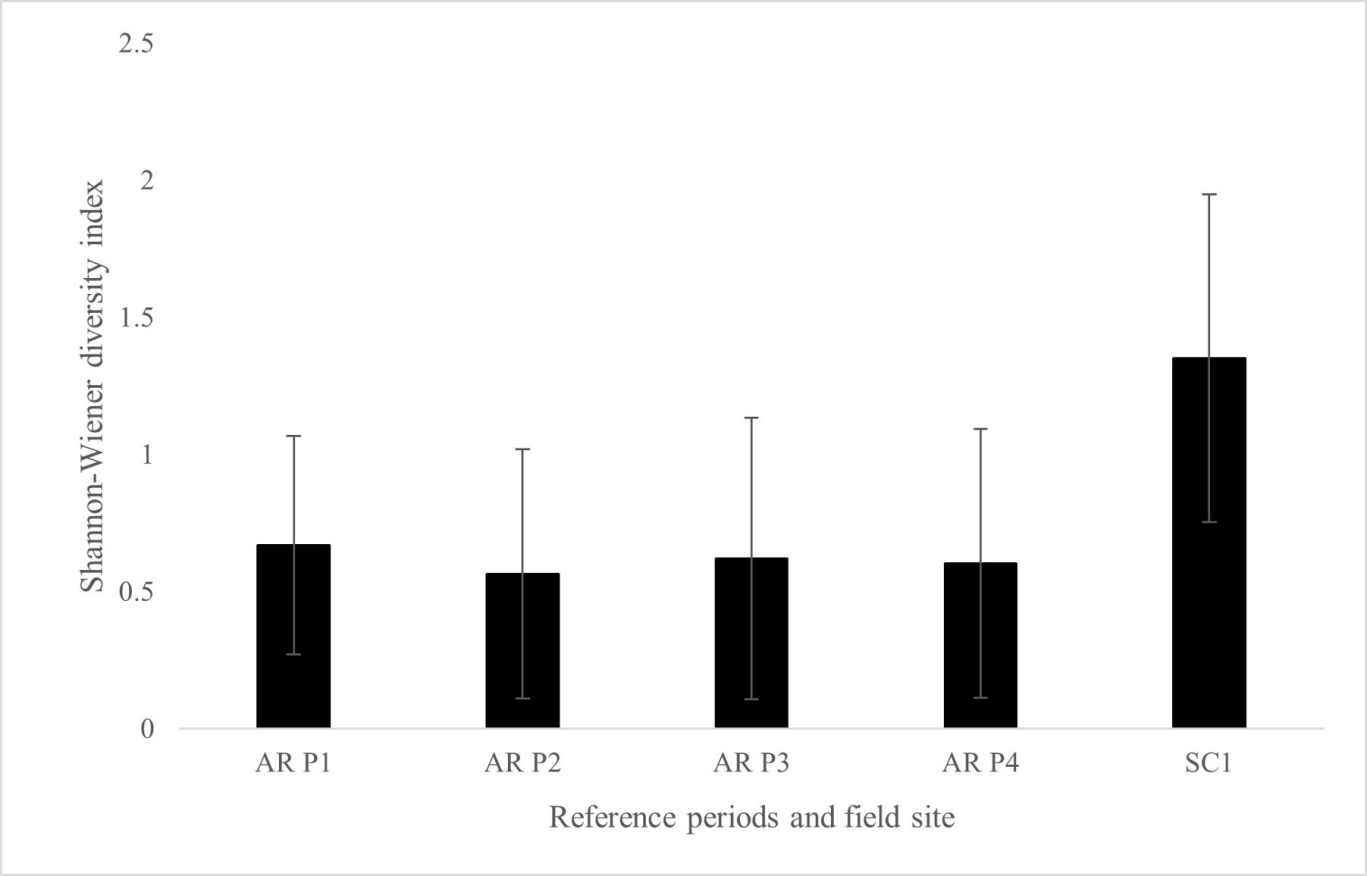
Average Shannon-Wiener diversity index for SC1 and reference periods. Average Shannon-Wiener diversity indexes for SC1 and AR reference periods (*p* < 0.01). SC1 different than all AR reference periods after *post-hoc* comparisons (*p* < 0.01).

**Fig 4:**
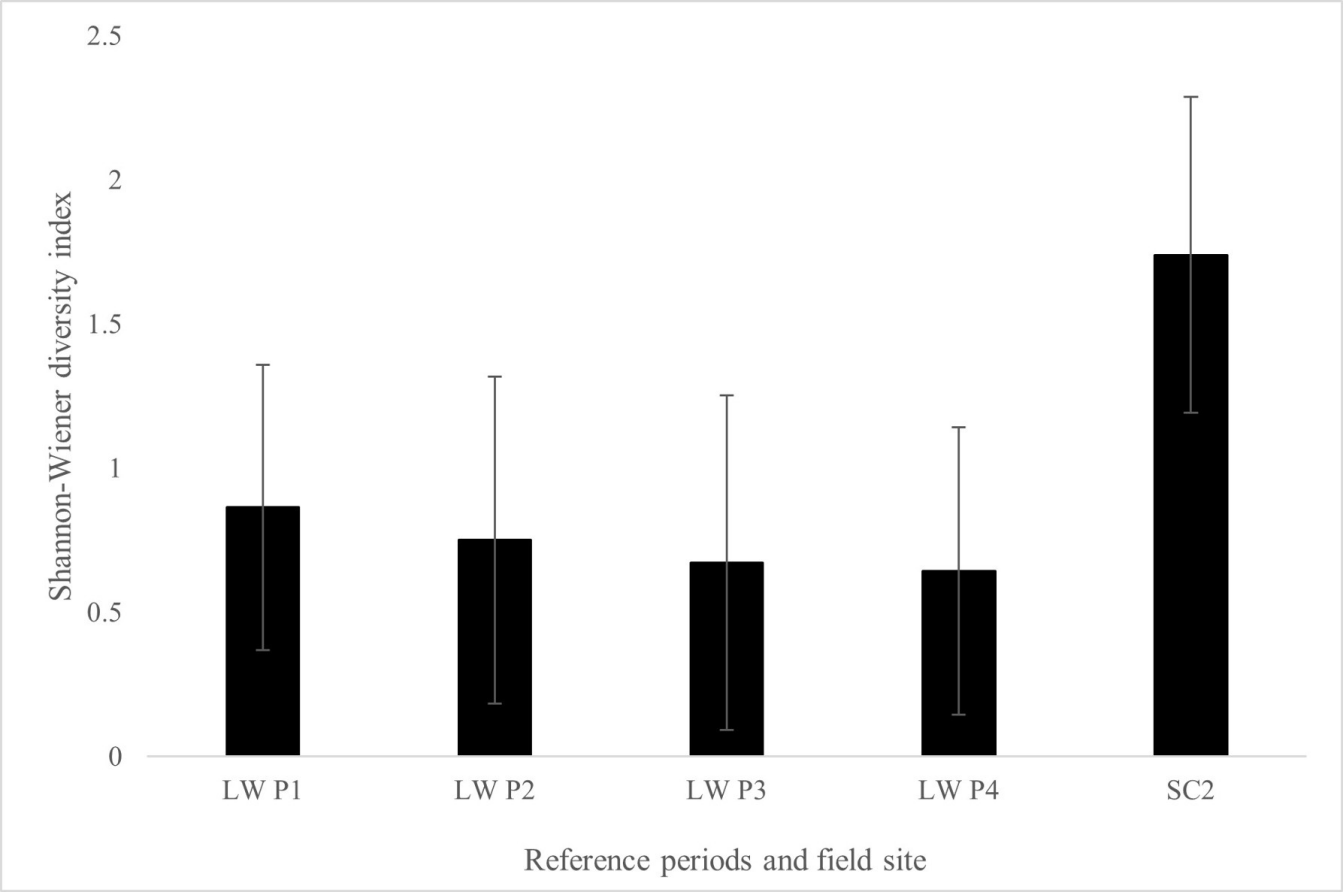
Average Shannon-Wiener diversity index for SC2 and reference periods. Average Shannon-Wiener diversity indexes for SC2 and LW reference periods (*p* < 0.01). SC2 different than all reference periods after *post-hoc* comparisons (*p* < 0.01).

Diversity was found to differ across periods at the North Carolina CC references sites (*p* < 0.01), while no differences across AR and LW reference periods were found. For SC1, differences of diversity (*p* < 0.01) and all AR reference periods were found (Fig 3). Differences of diversity (*p* < 0.01) were also found for SC2 and all LW reference periods (Fig 4). No differences of diversity at NC2 sites compared to CC reference periods were found.

After grouping species into functional guilds, differences of the proportion of benthivore species within SC1 and SC2 populations and their respective reference sites were found (Table 5). For SC1, 75% of the sampled population were benthivores which was more than the AR reference periods. While for SC2, 23% of animals fell within this niche which was less than LW sites. The proportion of epifaunal crustacivore species at SC1 and SC2 field sites were also different compared to their reference sites although overall, the proportion of animals found within this niche was generally low for both. For SC2, over 50% were pelagic planktivore species, greater than LW reference periods. Most species (96%) at NC2 fell within the generalist benthivores functional guild, similar to what was found at the CC reference sites (Table 5). For NC1, 83% of species sampled were pelagic planktivore species.

**Table 5:** Proportion of species within functional guilds for reference periods and field sites (totals = 1).

		*Benthivores*		*Epifaunal*			*Pelagic*			
Site	Period	1	2	3	4	5	6	7	8	9
AR	1	0.18^a^	0.66^a^	< 0.01^a^	0	0	0	0.1	0.06	0
	2	0.44^a^	0.36^a^	0.02^a^	0	0	< 0.01	0.06	0.12	0
	3	0.13^a^	0.29^a^	0.05	0	0	< 0.01	0.27^a^	0.25^a^	< 0.01
	4	0.09^a^	0.76^a^	0.03^a^	0.01	0	0.01	0.03	0.07	0
SC1	4	0.75	0	0.1	0	0	0.01	0.09	0.05	0
LW	1	0.56^a^	0.19^a^	< 0.01^a^	0	0	< 0.01^a^	0.02	0.21^a^	< 0.01
	2	0.39^a^	0.34^a^	0.06^a^	0	0	0.02^a^	0.02	0.15^a^	0.02^a^
	3	0.39^a^	0.13^a^	0.04	0	0	0.01^a^	0.01^a^	0.42^a^	< 0.01
	4	0.67^a^	0.05	0.26^a^	0	0	0.02^a^	0	0	0
SC2	4	0.23	0.07	0.04	< 0.01	0	0.59	0.03	0.03	< 0.01
CC	1	0.94^a^	< 0.01^a^	< 0.01	< 0.01	0	0.02	0.04^a^	< 0.01	< 0.01
	2	0.99^a^	< 0.01^a^	< 0.01	< 0.01	0	0.01	< 0.01	< 0.01	< 0.01
	3	0.96	< 0.01^a^	< 0.01	< 0.01	0	0.01	0.01^a^	0.02^a^	< 0.01
	4	0.83^a^	< 0.01^a^	< 0.01	< 0.01	0	0.16^a^	< 0.01	< 0.01	< 0.01
NC1	4	0.13	0	0	< 0.01	0	0.83	0	0.04	< 0.01
NC2	4	0.96	0.02	< 0.01	< 0.01	0	0.01	< 0.01	< 0.01	0

^a^Indicates significant difference between reference period and respected field site

No difference of skin, fin, and gill parasites or estimated total white blood cell counts across field sites were found (Tables 6 and 7). After analysis of differentials, a difference of location for eosinophils was found (*p* < 0.01) although averages for all sites were only about 5% compared to other differential white blood cells.

**Table 6:** Average ± SD ratings (0–3) for skin, fin, and gill parasitic loads across the field sites studied.

Location	Period	Skin	Fin	Gill
SC1	1	0.25 ± 0.61	0.29 ± 0.33	0.73 ± 0.77
	2	.	.	.
	3	0.06 ± 0.24	0	0.06 ± 0.24
SC2	1	0.08 ± 0.28	0.17 ± 0.38	0.22 ± 0.42
	2	.	.	.
	3	0.01 ± 0.12	0	0.04 ± 0.2
NC1	1	0	0.11 ± 0.33	0.22 ± 0.44
	2	.	.	.
	3	0.02 ± 0.13	0 ± 0	0.02 ± 0.14
NC2	1	0	0.29 ± 0.49	0.29 ± 0.49
	2	.	.	.
	3	0	0	0.04 ± 0.19

**Table 7:** Average ± SD total white blood cell counts and proportion of differential leukocytes across field sites.

	SC1	SC2	NC1	NC2
Total WBC	11890.32 ± 6765.36	11010 ± 7981.26	13717.95 ± 5895.05	13217.14 ± 5588.18
Lymphocytes	84.08 ± 11.91	80.92 ± 13.45	86.37 ± 8.35	81.92 ± 10.61
Neutrophils	13.04 ± 10.15	13.5 ± 12.1	12.09 ± 7.67	11.38 ± 6.41
Monocytes	0.88 ± 1.39	1.12 ± 2.19	0.54 ± 1.01	1.03 ± 1.52
Eosinophils	2.2 ± 4.23	4.42 ± 8.53	1 ± 2.16	5.67 ± 7.74
Basophils	0	0.04 ± 0.2	0	0

### Welfare assessments

**Fig 5:**
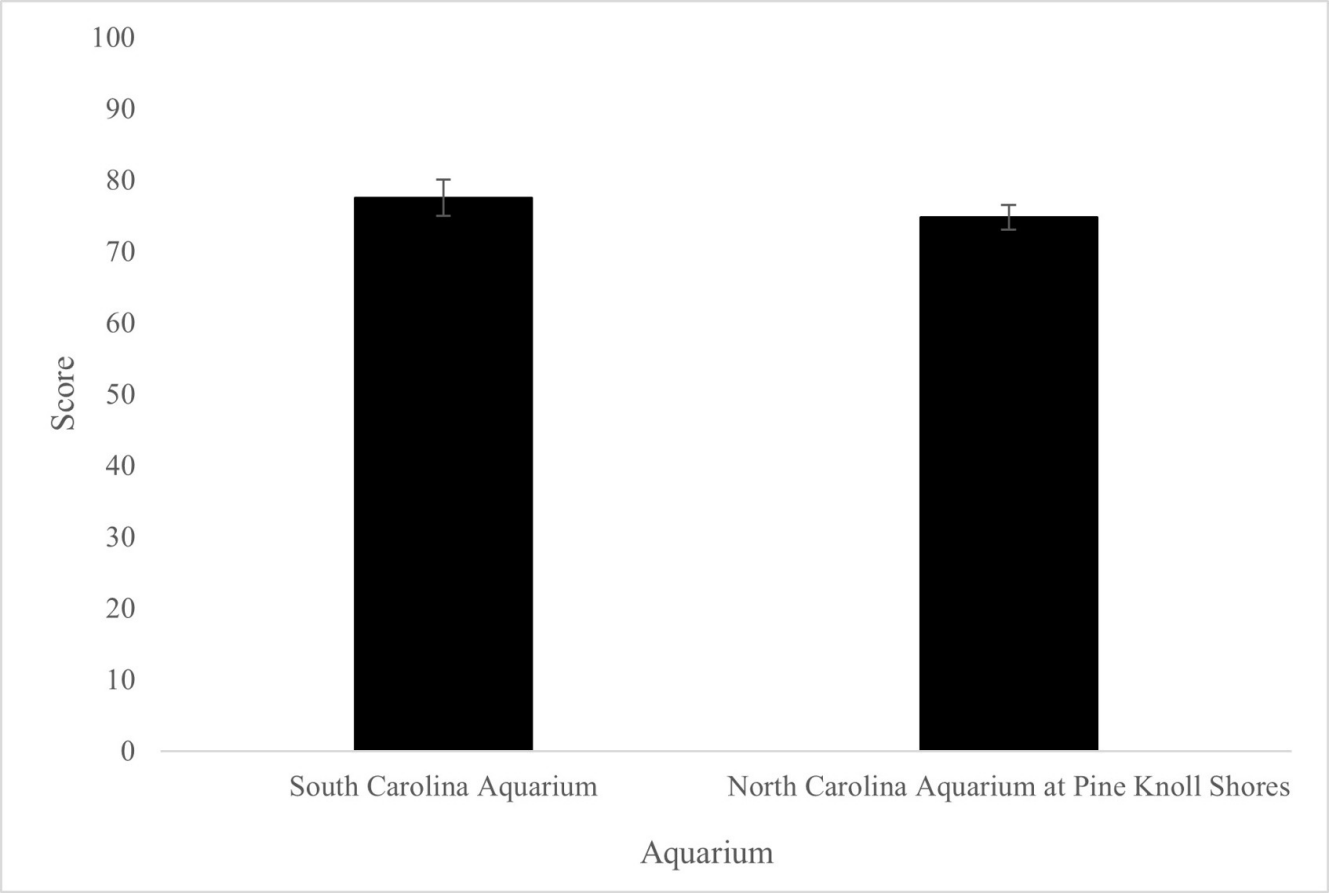
Average aquarium total welfare assessment score. Influence of aquarium on average total welfare score *(p* = 0.01). Ratings within each category of 1–3 (high risk–good) were summed and behavior ratings weighted by multiplying scores by two. Highest achievable score = 84.

Aquarium was found to influence total welfare scores (*p* = 0.01), while no influence of exhibit or period was found (Fig 5). No influence of origin (wild collected vs aquaculture) on average total welfare scores was found when comparing exhibits at the North Carolina Aquarium at Pine Knoll Shores (wild collected average with standard deviation = 74.8 ± 1.83, aquaculture average with standard deviation = 74.5 ± 2.12).

## Discussion

The objectives of this study were met by evaluating the ecosystem health of locations two public aquariums visit on an annual basis to collect fish for their institutions and then assessing the wellbeing of animals at each aquarium. Findings produced comprehensive assessments of each field site through measurement of chemical, physical, and biological indicators of ecosystem health. Evaluation of the wellbeing of fishes was achieved through measurements of indictors within the categories of nutrition, health, physical environment, and behavioral interactions. The hypotheses in this study were not upheld. Anthropogenic pressures of field sites were observed, but no evidence of high degradation or compromised health of animals were found. Welfare assessments of aquarium exhibit tanks produced high-positive scores overall, demonstrating that both wild collected and aquaculture fishes were coping appropriately within their environments.

In order to appropriately assess the ecosystem health of each field site, use of environmental and animal indicators was necessary for analysis of the influence of anthropogenic impacts, including public aquarium wild collection, on the functioning of each system [7, 14]. Measurements of water and sediment chemistries was an essential component of data collection as coastal ecosystems are sinks to industrial and agricultural pollution, and land use changes from tidal marsh to urbanized environments have occurred [2, 3, 58]. Differences of water parameters and reference periods were observed for some of the field sites but not others, therefore rejecting the first hypothesis (Table 3). Changes in salinity across reference periods was expected due to hydrological cycles and freshwater inputs, with dry years having higher salinities and lower salinities in years with increased rainfall [59]. Although differences were observed, all water parameters fell within the normal ranges for coastal environments indicating no immediate concern for animal or human health [60, 61]. For soil chemistries, potassium levels were found to be high at NC2 (73 kg/ A/15.24 cm soil), while all other parameters at field sites fell within low-medium ranges (18 kg– 36 kg/ A/15.24 cm soil). Previous studies have attributed high levels of potassium to wastewater runoff from agriculture, with excessive quantities leading to leaching in soil prohibiting nutrient uptake in plants [62]. Oyster restoration programs have been employed in many Mid-Atlantic coastal and inlet environments in efforts to restore degraded areas, although more data is needed to quantify the success of increased abundance on entire system function [63].

Incorporation of environmental assessments of each location provided key information about the biotic and abiotic characteristics of each habitat as well as the presence and intensity of human activities. NC2 scored highest out of all field sites due to this site having high complexity as well as being a protected area with no commercial or recreational pressures (Fig 1). Notable categories that scored 3/5 during assessments were morphology and complexity with evidence of erosion, instability, and moderate amounts of vegetative patches at SC1, SC2, and NC1 sites. Although these challenges are being met with active restoration programs, impacts of land use change within these systems should continue to be monitored. Previous studies have demonstrated that differences in intensity of human pressures influence the abundance and diversity of animal functional guilds living within these systems [8].

Use of fish as sentinel species in this study was important not only because they are targeted by aquariums, but because of their recreational and commercial role for humans, as well as ecological importance within the coastal systems they inhabit [3, 13]. Greater species diversity was found for both SC sites compared to their reference periods and therefore led to rejection of the second hypothesis (Figs 2 and 3). One limitation of this study was the difference of gear type used to sample fishes. Seine nets were chosen as the sampling method at field sites to mimic the sampling methods of both collaborating aquariums. Trammel nets, with larger mesh sizes capable of sampling larger size classes of fishes, were used at AR and LW reference sites while seine nets, for sampling juveniles, were used at all field sites [64]. For the CC reference sites, otter trawls targeted juvenile fishes, suggesting influence on the findings of lack of differences of diversity between NC2 and CC reference sites. Additionally, when comparing reference periods only, AR and LW showed no decadal differences of diversity and CC reference sites increased diversity overtime (CC reference period one average diversity with standard deviation = 0.99 ± 0.39, reference period two average diversity and standard deviation = 0.88 ± 0.33, reference period three average diversity with standard deviation = 1.06 ± 0.33, reference period four average diversity and standard deviation = 1.18 ± 0.33). Findings in this study also supported previous claims that communities within estuarine ecosystems contain a few species with high abundances and many species with low abundances [65–67].

Grouping species into functional guilds allowed for comparisons of the proportions of niches occupied within each system and considerations for how anthropogenic impacts may influence assemblages overtime. For SC1 and NC2 field sites, most species within the sampled populations were benthivores suggesting that increased inputs of runoff and pollution could cause substantial damage to the functioning of both ecosystems (Table 5) [9, 58]. For SC2 and NC2, most species were pelagic planktivores serving an important role as prey for larger species. High abundances within this functional guild suggests that predator populations may be declining, which could be attributed to increased anthropogenic stresses such as overfishing [47]. Additional research is needed to support these statements as potential shifts in functional guild assemblages across reference site periods was not overwhelmingly clear, apart from increases of pelagic species in AR reference period four (Table 5).

Health assessments of fishes was the final key component for use of these species as sentinels of ecosystem health at each field site as both a proxy and outcome of environmental health. Findings provided clear, timely reflections of how animals were responding to environmental changes resulting from anthropogenic impacts [68]. Parasitic loads at all field sites were extremely low with gill parasites being the most frequently observed (Table 6; SC1 avg. rating = 0.25, SC2 avg. rating = 0.08, NC1 avg. rating = 0.05, NC2 avg. rating = 0.06). Estimated total white blood cell counts showed no evidence of disease or compromised health in the animals sampled and differential counts fell within normal ranges (Table 7) [28, 69]. All these biotic and abiotic factors allowed us to a draw a metric-based conclusion that local harvesting pressures, including those of the aquariums assessed in this study, were not having a detrimental effect on localized ecosystem health.

Semi-annual welfare assessments at each collaborating aquarium allowed for evaluation of how wild collected fishes were coping within captive environments long-term. Previous assessments of the wellbeing of wild fishes shortly after arrival to aquariums while in quarantine showed animals’ ability to cope within newly restricted environments although instances of aggression were found in tanks containing multiple species [28]. Both institutions scored high overall, although aggression was observed while assessments were performed, demonstrating that some species may not be able to thrive in restricted mixed species environments. Evidence of aggression was the only behavioral indicator that scored high risk (1) consistently throughout all three periods of assessments, although aggression was not observed in the same exhibit each time.

The similarities of welfare scores between aquaculture and wild collected fishes demonstrated that aquaculture can be used to supplement wild collection of species. Further, the high scores assigned to both groups suggested that animals can cope in public aquarium settings long-term. This is a crucial finding in this study as previous assessments showed acute stress in commercial aquaculture fishes demonstrating a need for additional understanding of animal welfare in intensive systems and how animals purchased from this industry adapt in aquarium settings [28]. Additionally, sourcing from aquaculture brings the benefit of purchasing animals from controlled environments who are accustomed to being fed by humans and have received preventive care, including parasite management, prior to arrival to the aquarium and/or introduction to established tanks where they could cause devastation if harboring a communicable disease [70].

The collection practices of the collaborating institutions in this study proved to be low-moderate risk and did not detract from the functioning of the environment when compared to historical data. Due to the inability to separate aquarium pressures from other anthropogenic stressors at each field site, inclusion of animal wellbeing in aquariums was pivotal for considerations of shifting to increases in aquaculture instead of primarily collecting animals from the wild for these institutions in the future. More evaluations of aquarium collection practices, including larger institutions sourcing animals from fewer areas, are needed to determine if the findings in this study reflect public aquarium sustainability overall.

## Conclusion

Measuring ecosystem health is complex, but this study demonstrated that the South Carolina Aquarium and North Carolina Aquarium at Pine Knoll Shores are sourcing species from resilient field sites that can withstand the pressures of their current wild collection practices. Water and sediment chemistries were within normal ranges for coastal environments, environmental challenges were being met with active restoration programs, and populations sampled showed low parasitic loads and estimated white blood cell counts. Further, the welfare of wild caught versus aquaculture fishes were both positive and comparable. This study suggests that if done in low-moderate numbers, sustainable harvests can occur that do not detrimentally impact the environment or the health and wellbeing of collected animals, as compared to those sourced through aquaculture. Field site monitoring should be continued as anthropogenic expansion, and modification of, coastal environments has the potential to decrease system resilience. Additional research is needed to assess collection practices of other public aquariums as larger institutions with greater species numbers might impose greater stress on the systems they collect from.

As institutions that pride themselves on being leaders in conservation, education, and animal wellbeing, public aquariums are in the position to be leaders and influencers in improving the understanding of fish wellbeing in captive environments and advancing marine species aquaculture to take pressure away from stressed aquatic ecosystems. To achieve this, more research into species available through aquaculture is needed. Additionally, as many visitors to aquariums are themselves home hobbyists, these institutions hold a responsibility to advocate for the importance of sustainable sourcing of species, including purchasing through aquaculture. Through this study, we hope to influence additional assessments at other aquarium collection sites and increase resources for marine species aquaculture in the future.

## Supporting information

S1 Data (XLSX)
